# Feature selection for specific prediction targets at the user level in a district heating network

**DOI:** 10.1038/s41598-025-15777-0

**Published:** 2025-08-14

**Authors:** Samanta A. Weber, Michael Fischlschweiger, Dirk Volta, Jens Geisler

**Affiliations:** 1 Chair of Technical Thermodynamics and Energy Efficient Material Treatment, Institute for Energy Process Engineering and Fuel Technology, Clausthal University of Technology, 38678 Clausthal- Zellerfeld, Germany; 2 Energy and Life Science, University of Applied Sciences Flensburg, 24943 Flensburg, Germany

**Keywords:** Engineering, Energy infrastructure, Energy science and technology, Energy infrastructure

## Abstract

With the challenge of district heating network transition as part of the global objective of clean energy, machine learning provides a methodological approach for understanding the relationships between various influencing factors and demand-side properties of district heating networks, which is decisive for reducing losses, enhancing sustainability, and guaranteeing residential comfort. This work focuses on accelerating the application of modern machine learning methods to modeling district heating networks by generating knowledge on feature engineering and selection for newly suggested prediction targets, namely volume flow, supply, and return temperatures, directly at the building level. A systematic workflow for data acquisition, feature engineering, and selecting the most relevant predictors is presented. For this, statistical and machine learning methods are applied to engineer respective features and establish specific interdependencies, including meteorological conditions, human behavioral patterns, and operational parameters, based on a model region in northern Germany. The qualitative results indicate that the highest impact is for temporal predictors and operational features derived from the infeed facility’s data, i.e., approximately 15 to 20% of the total predictor relevance. In comparison to studies targeting the heat load and suggesting outside air temperature as the most relevant predictor, it was found that for the herein proposed prediction targets, this feature is of secondary relevance (roughly 6–10%). The findings of this study provide a feature engineering and selection strategy, as well as relevant knowledge gain, which is a prerequisite for efficient modeling of district heating networks based on machine learning in the future.

**Supplementary Information:**

The online version contains supplementary material available at 10.1038/s41598-025-15777-0.

## Introduction

Climate change-related phenomena have underscored the need for practical efforts in decarbonizing the energy markets. There should be increased interest in district heating networks, which are considered the most efficient heat supply strategy in urban areas^1–3^. In various engineering fields^4–7^, machine learning (ML) modeling strategies have been developed recently, offering distinct advantages in modeling complex problems and networks. Specifically, for district heating networks, the application of ML approaches could lead to increased automation^8^ and contribute to reaching the goals of carbon-neutral, sustainable, and efficient operation of district heating networks (e.g., Ref^2,9,10^. Yet, the information on the sophisticated choice of inputs, especially for modeling user-level parameters in district heating networks using ML, remains limited. Existing work focuses on predicting the heat load at the infeed facility^1,3,11–23^. A limited number of studies investigate the supply^24,25^ or return^26,27^ temperature as a prediction target. Only recently has the heat demand at user-level substations been investigated as a prediction target^28^. At the same time, findings emphasize the importance of integrating user data into the district heating networks’ control mechanisms to achieve efficiency gains^24,26,28–31^. With the potential for applying ML that the existing literature suggests, future research should provide the required knowledge, i.e., information on relevant features, for developing sophisticated ML models in district heating networks for user-level parameters.

The increasing effort to integrate ML methods into energy system operations highlights the relevance of research in this field. The selection of optimized inputs for an ML model is specific to the dataset. As a result, the process of feature selection is an essential part of building an ML model. The literature emphasizes the importance of selecting the most suitable predictors^13,32^. Furthermore, the relevance of selecting appropriate methods for feature extraction is stressed^13^. Inquiring about the most sophisticated feature selection process, existing work highlights the longevity and intense computational costs associated with feature selection, underscoring the benefit of access to domain-related results^11^. The proposition of additional features, which increase predictive information, is named as a relevant novelty^33^. However, in the case of district heating networks, the number of investigated predictors still tends to be limited, and further research is needed to increase the understanding of influencing factors^15,17^, especially for user-level parameter modeling.

This work focuses on accelerating the application of modern ML methods by generating knowledge on feature engineering and selection to investigate district heating networks for newly suggested user-level prediction targets, namely volume flow, supply, and return temperatures. The selection of the optimal set of inputs for ML models remains dataset-specific, with existing studies highlighting the relevance of feature selection. Yet, literature on the most sophisticated choice of inputs for ML user-level modeling in district heating networks remains sparse. Thus, this study proposes a systematic workflow for domain-specific feature engineering and selection, enabling the quantitative investigation of the relevance of influencing factors and derived features in novel prediction targets. This work utilizes experimental data from an existing district heating network in Germany as a model region for analyzing the interdependencies between operational, temporal, and meteorological parameters and the suggested prediction targets. Hence, the results are presented qualitatively, as they remain specific to this study’s model region until proven otherwise. This work relies on statistical and ML-based feature investigation and selection methods. As given by the well-established thermodynamic relationship for heat flow, which equals the product of mass density, specific heat capacity, volume flow, and temperature difference, the selected prediction targets convey thermodynamic information on the district heating network’s efficiency. Thus, understanding the output parameters at the user level increases knowledge for optimizing district heating network operation. Modeling user-level parameters with ML methods, therefore, can hold potential for enabling more effective control of district heating networks, reducing heat losses, optimizing load distribution, and facilitating the integration of renewable energy sources—ultimately supporting the transition toward more sustainable and carbon-neutral heating systems.

The article is structured as follows: Sect. 2 outlines the research methodology and input parameters, providing an overview of commonly selected features for heat load prediction. Domain knowledge and the literature provide the potential features for the suggested prediction targets. Further, the section introduces the model region and addresses the applied feature engineering and selection methods. Section 3 presents the case study and its results, which are discussed consequently. Section 4 finishes with the conclusions.

## Materials and methods

The suggested systematic feature engineering and selection approach is sequential: first, domain knowledge and literature research are used to identify the factors influencing district heating network parameters in the model region. The acquired raw datasets are then transformed into processable features through feature engineering. These form the set of potential features. Subsequently, the potential feature data are analyzed using statistical property visualization and correlation investigation to gain insight into the information content of the datasets and their interdependencies, thereby identifying redundant features. Consequently, feature evaluation and selection methods, both with and without the ability to utilize information from the sequential order of time series data, are applied to rank the relevance of potential features for the respective prediction targets. Lastly, by combining the information from the preceding steps, the results are interpreted to obtain a relevant and non-redundant subset of features for application in ML modeling.

### Model region

Feature selection begins with identifying the factors that influence the prediction targets. These factors are specific to the prediction targets’ datasets and, hence, particular to the model region presented in the following. The higher the comparability of a different model region to the one of this study, the higher the transferability of the results will be.

**Fig. 1:**
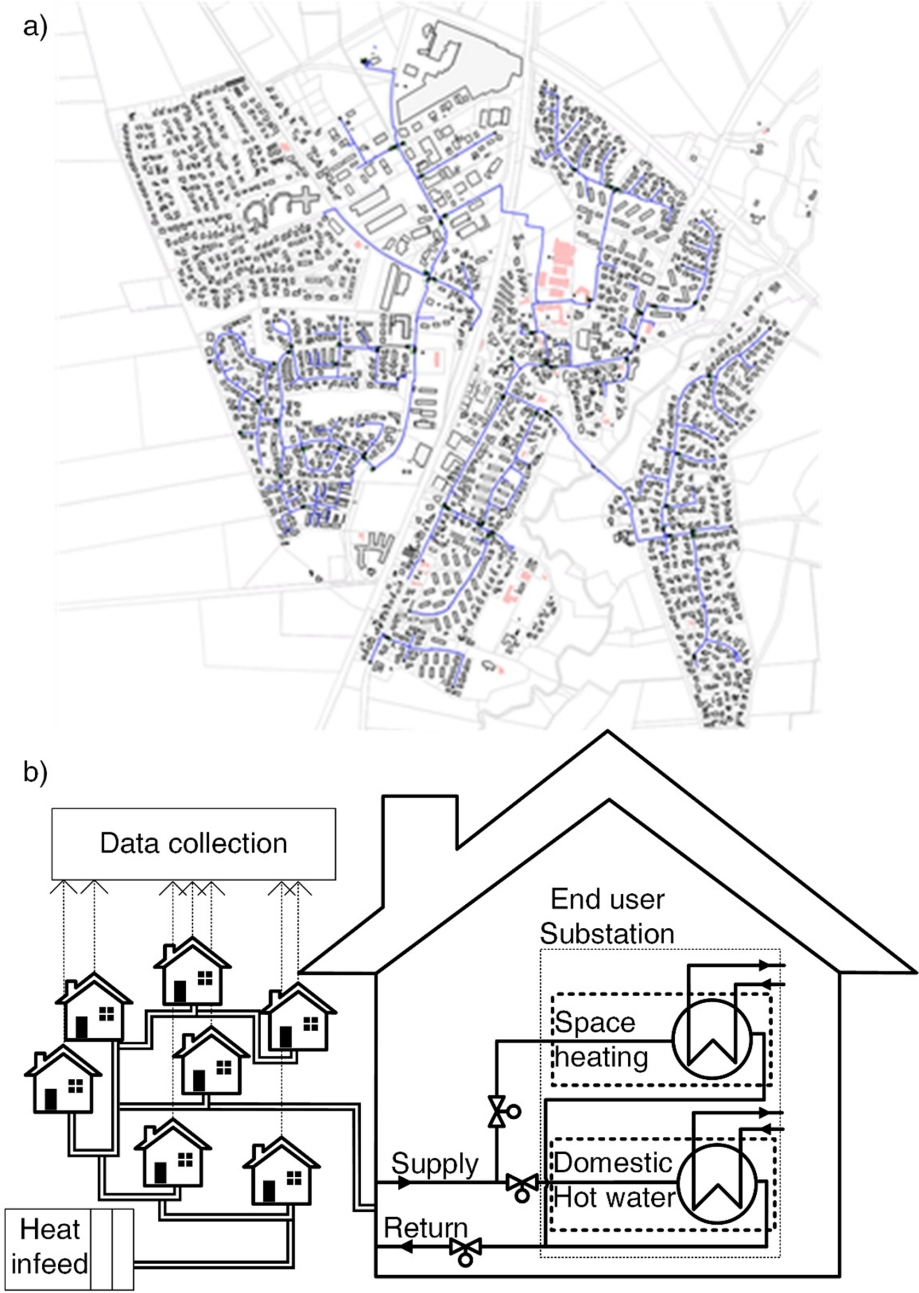
(**a**) District heating network piping structure, and (**b**) schematic heat transfer concept in the model region. The model region comprises a small, centrally supplied district heating network with mainly stub lines and one closed circle line as shown in (**a**). The figure (**a**) is reproduced with permission from Stadtwerke Flensburg GmbH. The users can receive both heat for domestic hot water and space heating, as presented in the schematic in (**b**).

Table 1 presents the technical parameters of the district heating network located in Tarp, Schleswig-Holstein, Germany, which is connected to a combined heat and power plant (CHP). Figure 1a displays the piping structure of the model region, and Fig. 1b schematically presents the network and heat transfer concept in the substations.

**Table 1:** Technical parameters of the district heating network in the model region.

Technical parameter	
Annual heat supplied	26 GWh
Peak load	12 MW
Operational supply temperature	70 to 105 °C
Return temperature	50 to 55 °C
Piping type	Plastic sheath pipes
Number of substations supplied	475

The supplied municipality, presented in Fig. 1a, consists mainly of stub lines, with one circular line also existing. As shown in the schematic representation in Fig. 1b, users in the model region can receive heat for both space heating and domestic hot water, resulting in year-round demand. Furthermore, domestic hot water can be supplied using a storage facility or directly, without the need for a storage tank. Deviating from Fig. 1b, it is also possible that the user receives heat solely for space heating.

Of the available network data, the recordings of the suggested prediction targets are collected directly at the substations. The recording of the substations’ parameters works remotely. The measuring unit sends momentary values approximately every hour to data accumulators in the network, making them digitally accessible. The address and the timestamp are identifiers. This study utilizes data on volume flow, supply, and return temperatures collected at approximately hourly resolution from substations from December 1, 2021, to June 17, 2023. For all substations, only 90% of the data is used for training and, where used, validation. Hence, the most recent 10% of the data is not used in any training or validation step. These are independent and remain to test the performance of a later model.

### Suggested prediction targets and influencing factor determination

For ML modeling, the influencing factors of the prediction targets must be converted into features, i.e., data that is processible by an ML algorithm. Thus, the starting point is to gain knowledge of the prediction targets. To better understand these, it is necessary to know that three basic operational modes can exist for substations, which can be distinguished by the volume flow recorded for each instance. The first operational mode occurs when there is no demand. Either the flow rate is very low for direct domestic hot water supply to maintain the supply temperature, or stagnation can occur in cases of only space heating supply or space heating and domestic hot water supply with a storage facility. If the system stagnates, the heat carrier in the connection line cools down in a non-stationary process. This volume must be replaced when the facility restarts. Hence, restarting is the second operational mode. The volume flow increases significantly, often reaching its maximum, as the supply temperature rises until it meets the required level, typically resulting in a high return temperature. Thirdly, the operation can achieve a relatively stable situation of a continuous, relatively stationary heat supply. The volume flow is adjusted to intermediate values. Visualization of statistical properties for the prediction targets, such as histograms and combination scatter plots, provides insight into their distributions and interdependencies. This information further contributes to determining the relevant influencing factors.

When a model for the substations’ parameters is established, the features must provide sufficient information to account for these operational modes. Thorough literature research provides the factors that influence heat load parameters. Regarding the dependency between the heat demand of individual buildings and the resulting overall heat load of the network, we assume a similarity between the respective influencing factors. Thus, Table 2 lists the predictors for district heating network modeling, providing an exemplary reference to related studies.

**Table 2:** Features for parameter prediction in district heating networks as given by literature.

Feature	Impact	References
Factors non-influenceable by operator		
Hour of the day	Use patterns over the day, heat capacities, control of heat exchange	^11,17,18,22,28^
Day of the week	Use patterns due to workdays and holidays	^11,13,22,25,28^
Day or month of the year	Seasonal impact, e.g., heating period and summer	^11,13,17,18,22,28^
Occurrence of a holiday	Impact on human behavior	^13,17,18^
Duration of measurements	Long-term effects	^13^
Outside temperature	Heat demand and losses of the buildings, perceived temperature	^11,17,22–24,28,29,31,33,42^
Ground temperature	Heat losses of the distribution pipes	^42–44^
Wind speed and direction	Perceived temperature, heat demand and losses of the buildings	15,17,23,27,29,31,33,42
Solar radiation, sun	Perceived temperature, heat demand and thermal gains of the buildings	^15,23,28,29,33^
Precipitation	Perceived temperature, thus heat demand and losses of the buildings, where applicable losses of the pipe system (moisture of the soil)	^23,31,43^
Air humidity	Perceived temperature, thus heat demand	^15,17,24,27,31,33^
Pipe diameter	Surface of heat transfer decisive for losses, resulting capacity flow impacts temperature loss; the heat capacity depends on the mass	^23,43^
Pipe length respectively geography	The longer the distance to the CHP, the higher the temperature loss in general; pipe length determines the surface of the heat transfer again and thus the losses	^23,43^
Insulation	The major impact on the thermal resistance should be located in the insulation	^23,43^
Return temperature	Necessary volume flow to meet heat demand, losses of the distribution system, efficiency of CHP	^14,31,42,44,45^ ^a^
Factors influenceable by operator		
Heat infeed	Over- or undersupply, system efficiency, return temperature	^14,31,42^ ^a^
Volume flow	Necessary temperatures to meet heat demand, losses of the distribution system, efficiency of CHP	^14,31,42,44,]^
Supply temperature	Necessary volume flow to meet heat demand, losses of the distribution system, efficiency of CHP	^14,31,42,44,45^ ^a^

^a^Measured at CHP, only.

This study aims to investigate the applicability of these influencing factors in the context of the new prediction targets. Domain knowledge provides insight into the applicability of the referenced influencing factors to the newly suggested prediction targets. Of the parameters listed in Table 2, this study employs the dynamic parameters only, apart from the coordinates, because ML algorithms, as used in this study, cannot derive the same information from static parameters as a physical model. Furthermore, this work does not introduce a linearly increasing feature to account for measurement duration, as it is expected to have low relevance for the suggested prediction targets. Having identified the influencing factors, these must be transferred into input datasets that are processible by an ML algorithm (“feature engineering”).

### Feature engineering for the influencing factors

The following addresses the process of developing representative features for the newly suggested prediction targets from the influencing factors. Certain influencing factors, such as weather parameters and operational data, can be described using measured values. Additionally, individual behavior reflects demand^29^. To express behavioral patterns as numerical inputs processible by ML algorithms, researchers employ values derived from the timestamp^11,13,17,18,22,25,28^.

The collected raw data requires transformation into processable and compressed information. To obtain relevant features, the data must be as clean as possible. Each time series demands individual pre-processing steps, which are adjusted manually. As newly suggested prediction targets are investigated, this study does not derive features by applying intense processing to the data, as performed in the literature (e.g., Ref^13^. Instead, this first approach relies on experimental characteristics.

Starting with the prediction targets, a resampling is conducted to achieve equal distances of hourly values using linear interpolation. Secondly, missing values are filled with a moving mean using a three-hour window to account for dynamics. After manually examining the data, the quality is assumed to be sufficient to omit outlier cleaning.

Subsequently, the operational and meteorological data demand adequate treatment. The data recorded at the CHP originates from internal monitoring and control of the facilities. Of the available data, the supply and return temperatures, the volume flow, the accumulated heat infeed, the supply and return pressures, and, in addition, the outside temperature form potential features. The cleaning of outliers is performed through filling and detection methods, as outlined in Table S1 (Supplementary Information). The differing dynamics of the features result in respective windows for cleaning, threshold value selection, and replacement values for outliers. Manually conducted visualization and testing of optimal choice of method determined the distinct selection of methods for detection, varying between mean, moving mean, and moving median. Table S1 (Supplementary Information) also provides the calculation rules for the latter. After cleaning the parameters of the accumulated heat, this study calculates the heat flow as the difference between consecutive hourly measurements.

Concerning the meteorological data, only the outside temperature is available at the location. Thus, this work utilizes data from the two geographically nearest weather stations, monitored by the German Meteorological Service (Deutscher Wetterdienst) [35] and located in Leck (1) and Schleswig (2), Schleswig-Holstein, Germany. Data quality information is available alongside the data. This study relies on the following data: ground temperature (1 m below the surface), minutes of direct solar radiation, wind speed and direction, absolute and relative moisture, as well as precipitation in hourly values. Firstly, this study equalizes the hourly timestamps with linear interpolation. Secondly, this work treats the missing values and outliers, as given in Table S2 (Supplementary Information).

Temporal values result directly from evaluating the timestamp. The occurrence of a holiday is transferred to a binary time series (0 not a holiday, 1 a holiday^17^. As for the coordinates, a uniform time series of the longitude and latitude, respectively, serves as input.

Furthermore, the data require scaling. Firstly, apart from the uniform time series and the occurrence of a holiday, all mean values of the signals are scaled to zero by subtracting the average. To equalize the peak-to-peak value of all signals to one and avoid the influence of magnitude, this work employs min-max normalization. Finally, any missing timestamps in the input or output data must be removed. For each substation, this leaves only the processable information.

### Statistical and correlation analysis for evaluating feature interdependencies

Visualization of statistical properties provides a first impression of the potential predictors’ datasets. It can indicate the information content of a feature based on the distribution and interdependencies of features, as shown in combination scatter plots. Features identified to show low information content can be expected to be assigned a low relevance in subsequent feature investigation and selection.

Further, feature redundancy should be addressed. Applying redundant features, i.e., features that provide overlapping or non-additive information to the model, does not increase the information contained in the feature set. Instead, this slows down computation and can lead to overfitting. Therefore, this study applies the standard method of linear correlation^14,15,29^ to quantify the linear interdependency of potential features. A numerical value indicating the intensity of the linear correlation between datasets provides an indicator of the difference in information content between potential features. Thus, a correlation investigation gives a means for identifying feature redundancy. Highly correlated features should not be applied in parallel. Instead, only the most relevant feature of the redundant should be selected. In contrast, a correlation between feature and target is indicative of high significance for the prediction. Non-linear mutual information is not captured in this step. This could include, for example, time-shifted occurrences, dynamic events, or non-linear correlations (such as exponential dependencies), among others.

Once the redundancies among the features are identified, the respective relevance of each feature for the prediction target shall be determined using the so-called feature selection methods.

### Feature selection methods

ML modeling architectures have diverse concepts. Therefore, the chosen methods for determining the feature relevance should reflect this. Thus, this study’s applied feature selection methods differ, especially in their respective approach to processing time series data, leading to distinct results. Specific ML algorithms enable the computation of information contained in the sequential order of time series data (e.g., recurrent neural networks, RNN). In the literature, the concurrent finding is that RNN structures, mainly the approaches of Long Short-Term Memory (LSTM) and deep neural networks, are most suitable for modeling thermal networks^1^.

Three general predefined approaches exist, namely filter, embedded, and wrapper type methods, for evaluating relevance and selecting representative features [36]. These are briefly explained below. Furthermore, the respective motivation for the selection of methods used in this study is provided. The feature relevance analysis will be conducted individually for each substation, and the results will then be aggregated (i.e., averaged) across all 475 substations to identify general trends. This approach was chosen to account for the heterogeneity of substation behavior while enabling conclusions regarding the features across the network. The numerical results for predictor relevance will be presented in descending order and subsequently categorized. When a significant difference occurs between two consecutive features in this order, a new category will be selected, ranging from features with the highest impact to those that are dispensable. This study applies MATLAB^®^ as the programming environment.

#### Filter type and embedded type feature selection

“Filter type feature selection” estimates the influence of a feature in terms of its relevance for the output using statistical measures^36^. Subsequently, the algorithm assigns a numeric value as an indicator^36^. For “embedded type feature selection”, the algorithm executes the feature importance estimation and the training of the model in parallel. Such algorithms evaluate the features based on the predictive power gained by the chosen model architecture from the inputs^36^. “Wrapper type feature selection” either encloses (forward) or withdraws (backward) features while determining the variation of performance a model achieves with the altered set of input parameters. Providing a termination condition, the model repetitively runs through training until no further improvement occurs^36^.

The applicability of the existing methods depends on the dataset, and not all existing methods are suitable. From the filter type, this study uses the principles of Maximum Relevance—Minimum Redundancy (MRMR), analysis of variance (ANOVA) with f-statistics (F-tests), and Neighborhood Component Analysis (NCA). MRMR searches for features that show the highest correlation with the target of prediction and, at the same time, the most substantial difference from the other features^37^. Reference^37^ presents the mathematical formulation. F-tests works with a hypothesis test, resulting in the p-value, which is smaller for a feature of higher influence. Any p-value smaller than *eps*(0) = 4.9407×10^− 324^ will result in an assignment of infinite feature importance^38^. The explicit mathematical formulation used in this study is presented in, e.g., Ref.^39^. The choice of these two methods relies on the general applicability to regression problems. Furthermore, all features are investigated in combination, and manual inclusion or exclusion is possible. A specific evaluation is provided as a numeric ranking. The NCA algorithm is based on a minimization method using the mean value of the leave-one-out loss as an objective function, as mathematically described in Ref.^40^. This work applies this method because it can capture the influence of correlation among the respective predictors with the leave-one-out approach^40^. The method evaluates the features that decrease the loss the most, while those that do not lower the loss enough are given zero influence^40^.

From the embedded type alternatives, this work applies the predictor importance of an automatically optimized regression tree (RT). This structure has the advantage of low computational costs. Furthermore, related studies^16,20^ utilize the RT modeling architecture for district heating networks.

The approaches from the filter and embedded type employed in this study do not consider the sequential order of the time series data as additional information. To reflect recurrent behavior as part of this study’s objective, an approach resting on an RNN is additionally applied. The conceptualization with a wrapper type feature selection method is explained below.

#### Wrapper type feature selection

The wrapper type feature selection algorithm requires a criterion function that provides a numeric value as a metric for the inclusion or exclusion of a feature. As RNNs are stated to be one of the most suitable choices for modeling district heating networks^1^, the aim is to test the integration of a recurrent structure into feature selection. In this study, the criterion function trains an RNN and subsequently evaluates the root mean squared error (RMSE) as a metric.

The chosen model must be well-balanced, meaning it is flexible enough to account for the dynamics and avoid underfitting, while also being sufficiently restricted in variance to prevent overfitting. A shallow RNN shall be applied to limit the computational costs. The hidden layer contains ten neurons. The network employs a layer delay of [1:2] to capture the influence of the preceding hour. The maximum number of 200 training epochs limits the process. Further selections of hyperparameters and settings of the ML model are captured in Table 3.

**Table 3:** RNN model hyperparameters and settings.

Hyperparameter/setting	Selection
Layers	Input, hidden, output
Units in hidden layer	10
Optimizer	Levenberg–Marquardt
Initial damping factor	0.001
Maximum validation failures	6

To determine the RMSE as the metric for feature selection, this study employs time series cross-validation (CV). The number of features corresponds to the number of groups to be specified for creating subsets of approximately the same size. The validation data must be sequential for time series CV and should be representative of the entire dataset. Literature on district heating networks indicates a significant difference in patterns between the heating period and summer^13,33^. Hence, two validation sequences are generated for each substation dataset originating from different seasons to minimize this influence on the validation results. This is achieved by setting the share of data to be selected for validation to the reciprocal of the number of features. Then, this amount is set aside for opposing seasons, beginning with January 1st and July 1st for the first feature, and then rolling out for the remaining features.

Before conducting any investigation based on the RNN, such as CV, it is essential to rule out model underfitting^41^. Therefore, the modeling architecture employed was investigated in this regard using the test data set that was set aside. To validate whether an underfitting of the model influences the performance, the autocorrelation coefficients of the residuals were investigated. A low autocorrelation of the residuals could be determined for an applied time lag. This indicates that the model captures most of the predictable part of the data. Rather than underfitting, the randomness of the data leads to the observed RMSE. Hence, the modeling architecture and CV appear to be applicable.

With this setting, the feature is selected if the addition (forward feature selection) to the set reduces the RMSE by at least 0.02. This metric value is experimentally adjusted and specific to our feature set. The overall RMSE differs for the individual buildings but ranges around 0.2. Hence, this study includes a feature if it reduces the RMSE by 10% of the expected value, which is a standard error threshold in significance tests^34^. Unlike the previously mentioned methods, this approach returns a binary list indicating whether a feature is included or excluded, rather than a continuous ranking.

## Results and discussion

The following illustrates the general distributions and interdependencies of the inquired information. First, a substation dataset is visualized to enhance the user’s understanding of the data. Second, when starting with the selection of the most relevant potential features, the meteorological data are exemplarily visualized. This highlights how the histograms and combination scatter plots provide an understanding of the information content and the interdependencies of the potential features. Third, linear correlation analysis outlines redundancies among the feature datasets. Lastly, using the gained insights from the previous steps, the feature investigation and selection methods provide a ranking of the quantitative relevance of predictors.

### Statistical properties’ visualization for the prediction targets

**Fig. 2:**
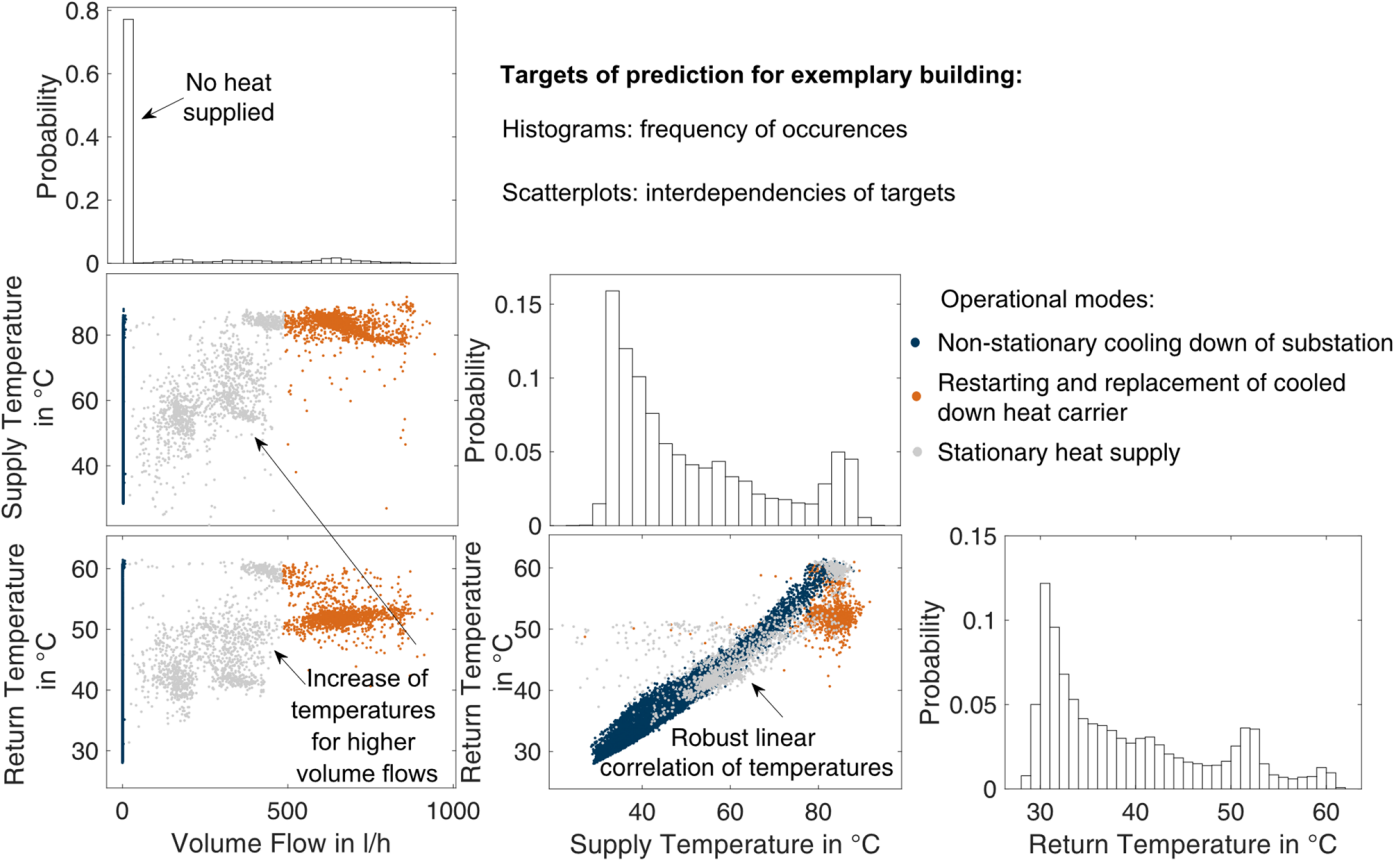
Histograms and combination scatter plots of prediction targets for exemplary building. The histograms of the prediction targets indicate more frequent occurrences. Displaying the data as scatter plots indicates a linear correlation between the temperatures and an increase in temperatures for higher volume flows.

To deepen the understanding of the prediction targets and the substations’ operation, Fig. 2 displays a histogram of the target in the column and a scatter plot of the target in the row against the column for an exemplary building. The choice of building does not rely on efficient operation, but rather on the suitability of visualizing the three possible operational modes addressed in Sect. 2.2.

To distinguish the operational modes, this work utilizes k-means clustering to separate the volume flow data into three distinct clusters. It applies the restriction that data with zero volume flow indicates stagnation, as the building has a domestic hot water storage facility, viz., the primary-side heat carrier can stagnate. The coloration of the scatter plots in Fig. 2 visualizes the operational modes.

As visible in the data on volume flow, a predominant majority of samples appear at a recording of zero, indicating times when no heat was supplied and limiting the amount of informative data for this target. For the remaining samples, a tendency for higher volume flow is observed, correlating with higher temperatures, indicating the system’s restart. Only a minimal amount of data appears for the stationary operation. The distribution of both temperature variables is skewed to the right and bimodal. Moreover, the supply and return temperatures display a robust correlation, particularly for lower values on both temperature scales, highlighting the internal relation.

It should be considered that a later ML model will be less accurate for occurrences with fewer data points, which can necessitate sampling measures. Additionally, specific statistical methods are sensitive to the skewness of distributions. Hence, the investigation also provides information for the intended modeling with ML algorithms.

Each substation’s data will be unique, resulting from influences such as storage capacities, supply strategies, and user behavior. Thus, the selection of features must contain sufficient information on all the relevant impacts for successful modeling. In the following, the features shall be investigated to determine a subset of relevant, but not redundant, features.

### Statistical properties’ exemplary visualization for the meteorological data

**Fig. 3:**
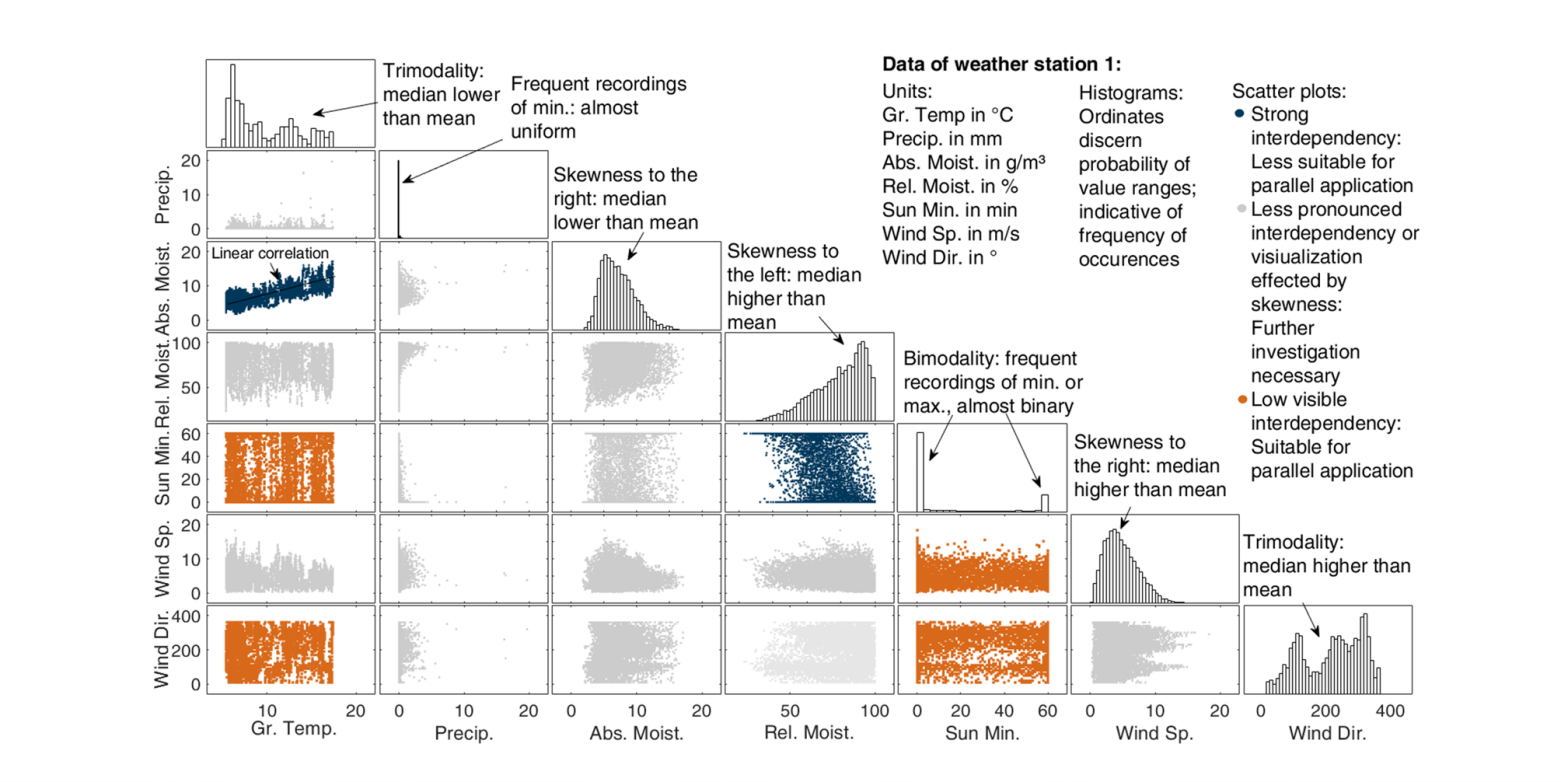
Histograms and combination scatter plots of parameters for weather station 1. Regarding the distributions of the meteorological data, the skewness indicates more probable ranges of values. Based on the scatter plots, a correlation is evident between ground temperature and absolute moisture, as well as relative moisture and sun minutes, while the other parameters appear to be less strongly related.

Visualization of statistical properties provides an initial impression of the information content of the features and potential redundancies. Exemplarily, Fig. 3 displays the histograms and scatter plots of the parameters for weather station 1. For better visualization, Fig. 3 abandons the titles of the histograms’ ordinates, which give the probability of the respective value ranges.

The distributions vary from right-skewed for absolute moisture and wind speed to left-skewed for relative moisture. The precipitation reveals a dominant peak at zero, indicative of high redundancy in the time series and associated low information content. This applies to the bimodal distribution of the sun minutes as well.

Evidently, the ground temperature and absolute moisture exhibit a robust linear correlation, suggesting one of the two is obsolete in modeling. To enable the quantification of existing redundancies, the following presents a correlation analysis of the features.

### Correlation analysis of the features

**Fig. 4:**
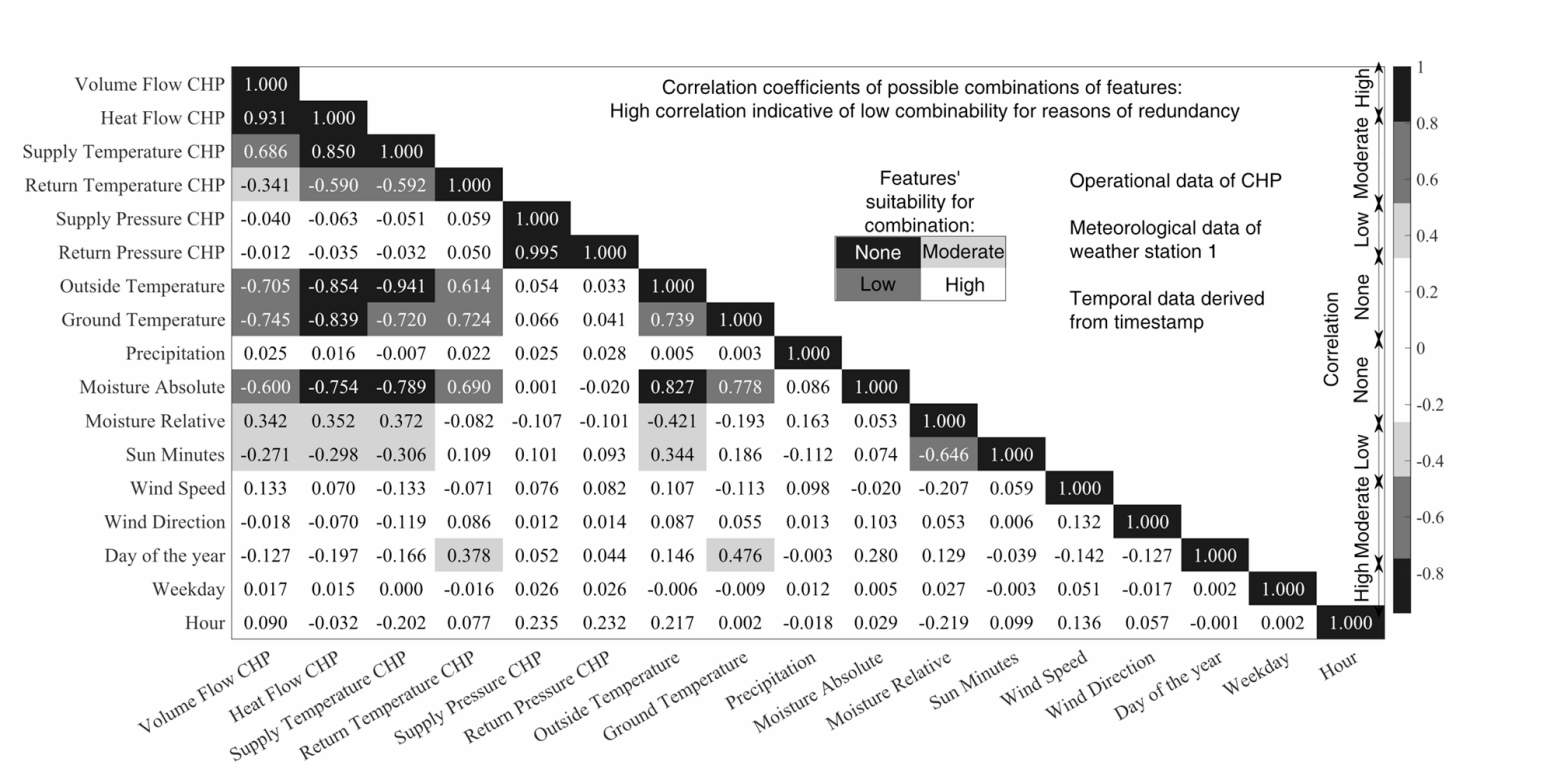
Evaluation of feature combinability applying linear correlation. The correlation coefficients indicate linear dependencies by very high or low values. If two predictors exhibit a high correlation, they should not be applied parallelly for ML models.

Figure 4 displays all possible combinations of two potential features, stating the correlation coefficients, as well as the ranges for evaluating correlation intensity^15^ and the resulting evaluation of the suitability of two features to be applied parallelly.

The highest values (dark grey) in Fig. 4 correspond to the correlation of operational data, including supply and return pressure, heat and volume flow, and heat flow and supply temperature. Regarding the weather parameters, the ground temperature and absolute moisture mutually depend on the outside temperature. Furthermore, the operational return temperature correlates with the three named weather parameters.

The operational supply temperature, used as a control variable, relies on the outside temperature, resulting in a robust inverse correlation, as indicated by the negative values. Less pronounced, the operational heat and volume flow also exhibit this interdependency. Based on mutual causality, the same applies to the three operational parameters, combined with the ground temperature or absolute moisture. Additionally, the sun minutes and relative moisture appear to be moderately related (lighter grey colors). Furthermore, the combination of the day of the year with the ground temperature and operational return temperature exhibits moderate correlation coefficients, particularly as the return temperature increases towards summer. The remaining factors showing no correlation (white) are potentially independent. Regarding the time series of coordinates (constant values) or the holiday (binary time series), the correlation does not apply, which is why they are excluded from Fig. 4. Since the evaluation is limited to linear correlations, other types of mutual information are not accounted for.

To investigate the applicability of the alternative meteorological data’s locations, we conduct a correlation of the two weather stations’ recordings, as displayed in Fig. S1 (Supplementary Information). As all parameters, apart from precipitation, correlate moderately to highly, we assume the same for the third location of the model region situated between. Consequently, only the precipitation can potentially not be accounted for. Due to small differences and high computational costs, only the data of weather station 1, located upwind in the main wind direction, is used in the following evaluation. The redundancies among the features are now outlined. However, the relevance of the features for the prediction targets still needs to be determined. Therefore, this study applies feature selection methods to determine the most relevant set of inputs hereafter.

### Feature evaluation with MRMR, F-tests, predictor importance RT, NCA

Firstly, an investigation of the feature set is conducted using the explained filter and embedded type methods. This work interprets the results by employing MRMR, F-tests, predictor importance analysis based on RT, and NCA for the entire dataset. The results are divided by the respective sum to normalize the total of all predictor rankings to 100%. For the F-tests, this study substitutes any infinite values with the maximum value in the respective ranking prior to normalization. Averaging over the individual buildings’ results gives the mean value for the respective target of prediction.

The seasonal differences existing in district heating networks^13,33^ eventually demand special treatment. To test the influence of such differences on the novel prediction targets, Fig. S2 (Supplementary Information) depicts the results of the separate analysis. For computational efficiency reasons, this study only employs MRMR for this specific investigation. The exhibited discrepancies confirm the need to capture seasonal behavior. Yet, no input parameter would be excluded for one season, which is decisive for the other. If the model architecture cannot account for long-term behavior, it could be more suitable to work with separate predictive models for the summer and the heating period.

**Fig. 5:**
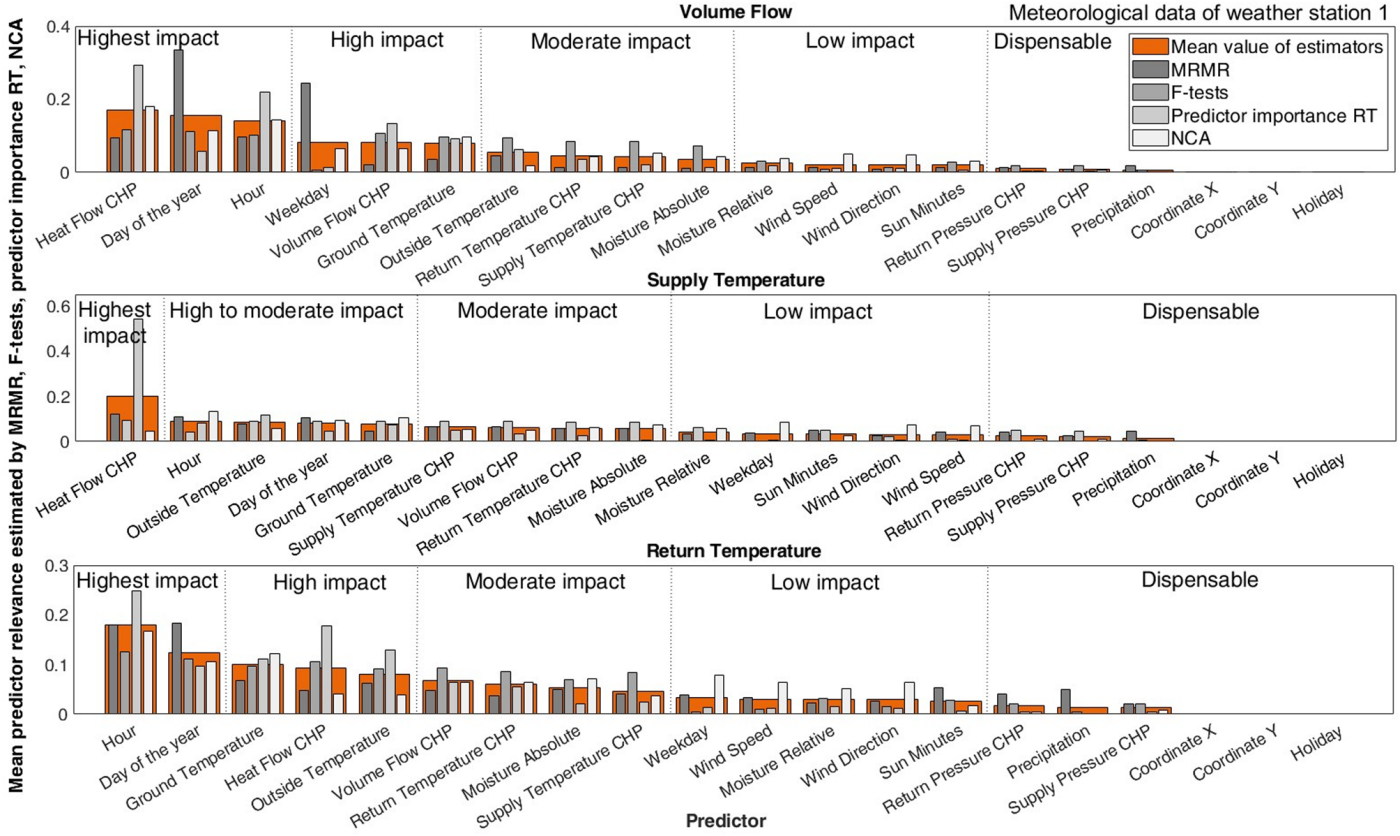
outlines the final results as the mean of buildings. None of the algorithms reveals any relevance from the coordinates or the holiday time series, which is why they are not discussed further.

Figure 5 Concluding results for MRMR, F-tests, predictor importance RT, and NCA. Regarding the overall results of the predictor evaluation, the general order of relevance is congruent, while the explicit results of the methods differ. Each target of prediction exhibits a respective ranking of relevance.

The specific order of relevance differs for the prediction targets. Hence, the following sections interpret the results for the prediction targets separately. Subsequently, Sect. 3.4.4 outlines the results generalized over all prediction targets.

#### Volume flow as prediction target

Starting with the volume flow, the heat flow at the CHP ranks highest with 17.05% of the total feature relevance. Literature confirms the high impact of the operational parameters^31^. Furthermore, the very relevant influence of the temporal values of the hour of the day (15.50%) and the day of the year (14.05%) is evident. Again, the integration of temporal factors by a considerable number of researchers underscores the common conception of their relevance^11,13,17,18,22,25^. For the volume flow at the building substations, the temporal values being among the features with the highest impact suggests that human behavior or control mechanisms based on temporal indicators have a strong influence. In comparison, the weekday (8.21%) ranks considerably lower, indicating less influence.

The algorithms place the volume flow at the CHP next in order, with 8.19%. MRMR and NCA estimate the operational heat and volume flow with a lower influence than the other two algorithms, underlining these features’ common causality. This finding concurs with the necessity of eliminating one of these predictors, making the volume flow less relevant.

References^17,18^ rank the factors influencing the heat load that they investigated, determining the outside temperature as the most critical factor. However, regarding the ground and outside temperature, following in the ranking with 8.06% and 5.45%, only MRMR would lead to preferring the latter. Concerning the remaining meteorological data, apart from NCA, none of the evaluations would include wind speed (2.08%) or direction (2.05%). Evidently, the operational pressure values, as well as the recordings of the sun minutes and the precipitation, which range between 1.00% and 0.62%, show a very low interdependency with the volume flow at the building.

For the volume flow as a prediction target, the information from the correlation investigation and the feature ranking shall be combined: the outside temperature and absolute moisture, as well as the volume flow at the CHP, should be considered for exclusion due to high correlation. The pressure values at the CHP, alongside the sun minutes and the precipitation, could be excluded because of low predictive relevance. The heat flow at CHP and temporal predictors have the highest impact on the volume flow.

#### Supply temperature as prediction target

Emphasis should be placed on the findings relating to the supply temperature, as this prediction target has a guaranteed limit not to fall below and is decisive for network losses. By modeling the supply temperature accurately enough, safety margins might be slimmed, resulting in overall more efficient operation.

Remarkably, the most important influencing factor concurringly appears to be the heat flow at the CHP (19.94%), with the RT placing a significantly high impact of 54.20% on it. The hour of the day, outside temperature, day of the year, ground temperature, and operational supply temperature follow in order, accounting for 9.07–7.70% of the total predictor relevance. Although the outside temperature is considered very important by researchers for the heat load as the prediction target^17,18^, other features surpass its relevance for the suggested prediction targets, and the specific model region investigated in this study. Considering Fig. S2 (Supplementary Information), the outside temperature shows a peak for the supply temperature in winter, meaning it is more relevant in the (comparatively longer) heating season.

In descending order, the volume flow at the CHP follows, reaching 6.04%. Yet again, the heat flow at the CHP exhibits a higher influence. In comparison to the results for the volume flow, the weekday ranks much lower (3.28%).

Hence, the ground temperature and absolute moisture, as well as the volume flow at the CHP, seem less suitable for modeling because of their high correlation. The wind speed and direction, as well as the pressure values at the CHP, and the precipitation, also seem insignificant, with values of 3.15–1.29%. The supply temperature relies mainly on the heat flow at the CHP.

#### Return temperature as prediction target

Looking at the return temperature, the pattern of the first predictors in the ranking remains roughly the same. Predominantly influenced by the hour of the day (18.05%) and the day of the year (12.44%), the results emphasize the influence of the temporal variables again, even more than for the other two targets, yet placing a significant role on the ground temperature (9.95%) and the heat flow at the CHP (9.28%). MRMR finds more predictive power in the temporal variables, resulting in lower values for the remaining features. Furthermore, this algorithm detects a significant relevance of the precipitation (5.05%). Also, the RT suggests considerable relevance of the hour of the day (24.87%). Concurringly, the ground temperature ranges before the outside temperature and absolute moisture. Only NCA places higher relevance on the weekday (7.86%), as well as the wind speed and direction and the relative moisture (6.50–5.07%), which indicates their low redundancy.

Therefore, the pressure values at the CHP and the precipitation appear obsolete because of their low significance (1.78–1.34%). However, the volume flow at the CHP, the outside temperature, and absolute moisture should be investigated further to determine their relevance because of their high correlation. The return temperature is primarily influenced by the temporal values, and the ground temperature.

#### Feature selection with MRMR, F-tests, predictor importance RT, and NCA

The evaluation underlines the highest influence of the temporal features and the heat flow at the CHP. The high relevance of the ground temperature for the chosen model region emphasizes the individuality of feature relevance depending on the prediction target. The results for the suggested prediction targets differ from the results for modeling the heat load, for which the common notion^11,17,22–24,27,29,31,33,42^] states a supreme relevance of the outside temperature, which is often the only considered external input^11,25^ and claimed^29^ or even ranked with feature investigation methods^17,18^ to be the most significant factor. At the same time, only a few studies integrate the ground temperature^42–44^. Yet, the findings indicate that for both targets of volume flow and return temperature, the ground temperature holds more predictive power than the outside temperature.

The following compares the results obtained so far to those of the approach using an RNN with wrapper type feature selection to capture the influence of the information contained in the sequential order of the time series data.

### Feature selection applying RNN for considering Temporal dynamic influences

**Fig. 6:**
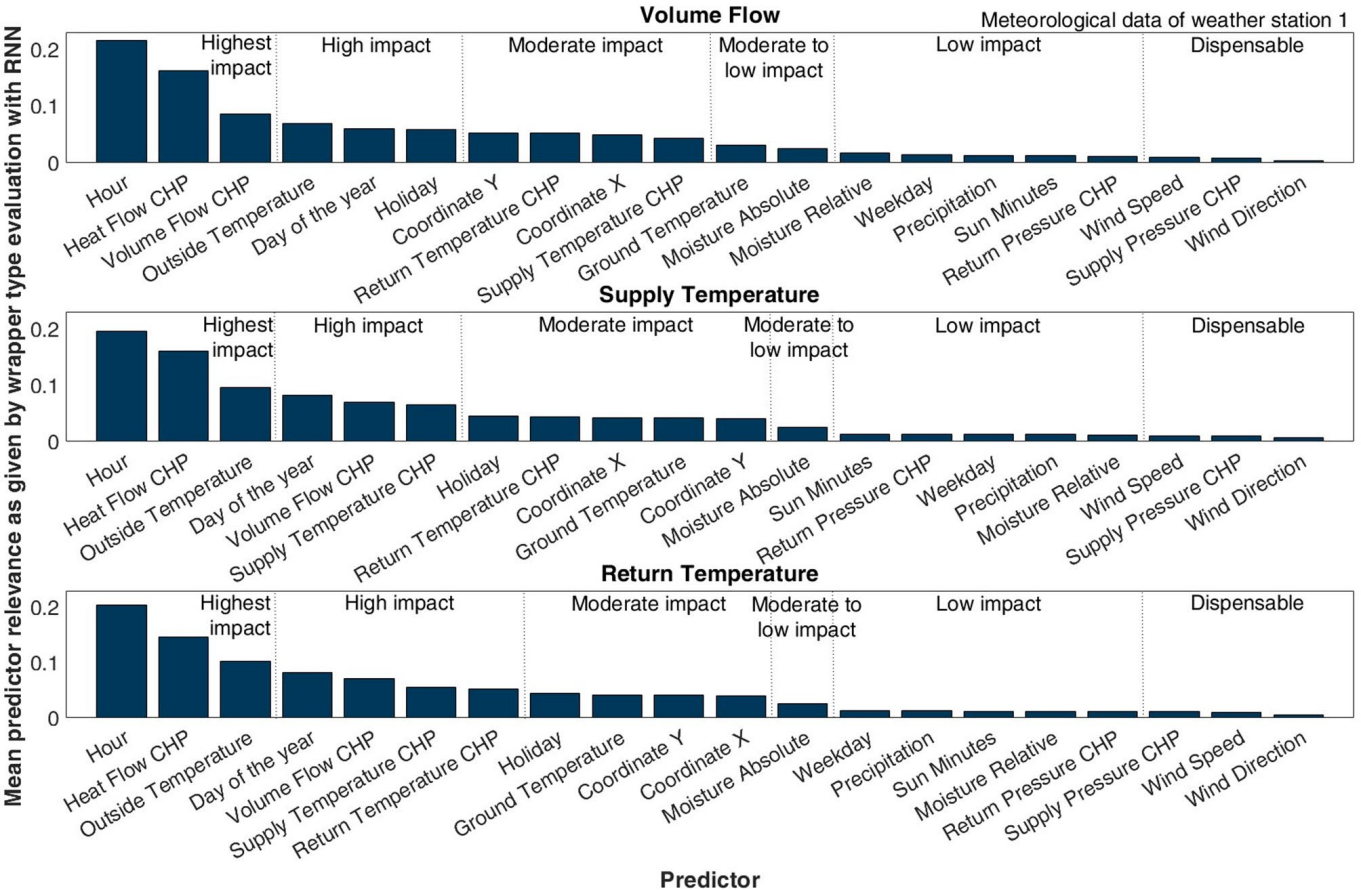
Predictor ranking using RMSE of RNN for criterion function. The approach relies on a recurrent structure, enabling the factoring in of a certain degree of dynamics, which results in a change in the relevance of specific features compared to the former analysis. Yet, the highest impact of temporal variables and the operational data remains valid.

Figure 6 displays the resulting ranking by applying the wrapper-type algorithm in combination with an RNN described in Sect. 2.5.2.

For all prediction targets, the hour of the day (21.61–19.58%) and the heat flow at the CHP (16.24–14.69%) exhibit the highest impact. The remaining features with highest or high impact differ for the prediction targets in descending order. Still, they concurringly include the volume flow at the CHP (8.62–7.05%), the day of the year (8.27–6.06%), and the outside air temperature (10.31–6.87%). The differences in relevance between the prediction targets for the features with moderate to low impact and the dispensable features are minor but result in specific descending orders. Confirming the preceding findings, the operational volume flow is assigned a high relevance, but it is lower than that of the heat flow at the CHP. Additionally, in line with previous findings, the weekday has a lesser impact than the other two temporal values. Concurring with the earlier statements, neither the pressure values at the CHP nor the precipitation significantly influence the prediction targets (1.31–0.55%). However, the wind speed and direction are found to be at the lower end of significance for this evaluation, meaning the RNN can gain even less information from them than the first set of methods. The relative moisture also provides less information in this setting than for the first set of estimations.

Differing from the first investigation with non-recurrent algorithms, the hour of the day clearly achieves the highest rankings, indicating that the recurrent structure detects more influence in this temporal variable. In contrast to the non-recurrent analysis, the outside temperature surpasses the ground temperature (e.g., for the supply temperature as prediction target 9.55% vs. 4.24%) and clearly holds high relevance. Evidently, the recurrent structure can gain more information from the comparatively more dynamic outside temperature than the set of methods in the first investigation approach. Additionally, the results for the temperatures of the ground and the air differ, which can be explained by the distinct characteristic of the feature selection methods used. As the ground temperature tends to exhibit slower temporal variation, it may capture the baseline thermal conditions affecting buried infrastructure and heat losses in underground distribution pipes more adequately. This could explain its higher relevance in static feature selection methods that prioritize stable, long-term patterns. In contrast, the RNN model may process short-term temporal dynamics more successfully and therefore benefit more from the rapidly changing outside air temperature, which directly influences short-term demand fluctuations, occupant behavior, and heating control responses.

Moreover, the RNN appears to be able to account for the influence of the holiday (5.93–4.47%), indicating that this input should not be neglected when using recurrent structures for modeling. The coordinates are integrated as a uniform time series. An RNN can account for an offset with the bias, but the model still selects these predictors. It is concluded that a uniform time series of ones could be used as an additional feature depending on the choice of modeling architecture. As the other results, in essence, follow the conclusions already drawn, the sequential feature selection confirms the remaining choice of inputs.

### Summary and discussion

In combination with the correlation analysis results, the features are in-/excluded as given in Table 4. A reason for the in- or exclusion is stated for each feature. Certain additional aspects should be considered, as discussed below.

**Table 4:** Selection of features based on results of feature evaluation methods.

Feature	Reason for in-/exclusion
Heat flow CHP	Necessary for closing the loop between operator and building, high correlation with volume flow CHP, chosen for higher rankings
Volume flow CHP	Comparatively high correlation with heat flow at CHP, but achieves lower rankings
Supply temperature CHP	Necessary for closing the loop between operator and building, but high correlation with heat flow at CHP and outside temperature
Return temperature CHP	Necessary for closing loop between operator and building. Relatively low impact but not dispensable
Supply pressure CHP	Redundant due to almost exact correlation with return pressure, no relevant influence determined
Return pressure CHP	Redundant due to almost exact correlation with supply pressure, no relevant influence determined
Outside temperature	Higher dynamics than ground temperature, decisive for supply temperature at CHP, yet redundancy with ground temperature, importance for supply temperature more pronounced, higher relevance for application of recurrent neural structure
Ground temperature	Decisive for losses of the system, lower dynamics than outside temperature, redundancy with outside temperature, higher relevance for application of statistical model
Absolute moisture	Strongly correlated with outside and ground temperature, but not dispensable
Relative moisture	High correlation with sun minutes, but achieves higher rankings, small influence in general, determined as dispensable using RNN
Sun minutes	Increased importance in summer, therefore neglection regarded as critical for this season, despite low rankings overall
Wind speed	Low rankings overall, determined as dispensable using RNN, test for recurrent algorithm architectures with long-term memory, as decisive for dynamics of weather phenomena
Wind direction	Low rankings overall, determined as dispensable using RNN, test for recurrent algorithm architectures with long-term memory, as decisive for direction of weather phenomena
Precipitation	Excluded for means of low relevance. Possibly, low rankings due to low applicability to location of model region and highly skewed distribution. MRMR suggests higher relevance, only
Holiday	Applicable for recurrent networks
Day of the year	High predictive power, therefore considered decisive for modeling of seasonal impact
Weekday	Important for users with highly constant patters (industrial, residential complex), more predictive relevance for summer
Hour of the day	High predictive relevance, therefore considered decisive for modeling of human behavior
Coordinates	Transfer to one uniform time series input

Firstly, the quality and quantity of the data influence the results of our study. The primary objective of applying ML is to identify the general pattern in the data, without relying on noise. Notably, the heat flow at the CHP was found to exhibit higher predictive power than the correlated volume flow at the CHP. The operational heat flow, as described, is calculated using the cumulated heat, a dataset that can be cleaned very effectively, as the value should only increase. Thus, the data on the operational heat flow may contain less noise than that of the volume flow at the CHP, even though both values undergo a cleaning process. In the same context, it is possible that the precipitation recordings do not match the location of the model region. The results must be understood under this restriction. Yet, under the assumption of adequate data collection, the general findings should be independent of this circumstance, such as the highest impact of temporal and operational predictors compared to the meteorological data.

Furthermore, the results of this research indicate the relevance of distinguishing between the summer and heating periods. Again, this finding is specific to the location investigated.

Moreover, the list of investigated influencing factors cannot claim completeness because the findings for the newly suggested targets can be cross-referenced only to a limited extent. Based on our work, research can focus on further developing the list of influencing factors and determining the most suitable manner of expressing their impact in numerical terms. It is also possible, given that the targets appear to be related, to consider using one target as input to the other.

Of the selected algorithms for feature investigation, only one relies on a recurrent structure. In this first example, our study employed a simple structure of a shallow RNN. Thus, it would be interesting for future studies to employ such architectures as well.

Aiming to integrate ML models for district heating networks, our work provides a starting point for modeling individual substations. If the black-box architecture and dependencies of the data return a model that is robust enough, the framework could be transferred to other district heating networks. If adequate accuracy can be achieved, an increase in system efficiency may become possible by slimming margins and reducing the need for a physical digital twin model. While ML models offer the potential for automation of the model-building process^8^, they may lack the robustness and interpretability that physics-informed models provide, particularly in safety-critical or extrapolative scenarios [46]. Thus, future research could investigate the most sophisticated level of integrating physical information and automating model building using ML.

Most importantly, until proven otherwise, it must be assumed that the findings of this study remain applicable only to the model region. Naturally, the size of the thermal network, along with its age, maintenance status, connection rate, demand density, and meteorological conditions, as well as social structures, affect the individual results of each district heating network in question. Thus, this study aims not only to provide information on the chosen model region but also to increase knowledge of district heating networks in general by identifying influencing factors and outlining a feature investigation workflow. Future research comparing the explicit findings of this study to those of other model regions would increase the benefit.

## Conclusions

This study aims to accelerate the application of ML in district heating networks by determining the required knowledge on feature engineering and selection for newly suggested user-level prediction targets, namely volume flow, supply, and return temperatures. It employs statistical and ML methods to investigate the feature engineering and selection process at the building level for a district heating network in northern Germany. The study first determines influencing factors and engineers relevant features from the raw data. Second, this work identifies redundancies among the features and assesses the relevance of each feature. The objective is to select a subset of non-redundant, relevant features for ML modeling purposes. This study further compares statistical and ML-based feature evaluation and selection methods with a feature selection algorithm based on an RNN to assess the impact of incorporating additional information from the dynamic behavior contained in the sequential order of the time series data.

A significant finding is the influence of temporal predictors and operational data from the infeed facility. Notably, specific temporal features, i.e., the time of day and the day of the year, have the highest impact on the suggested prediction targets, accounting for approximately 15–20% of the total predictor relevance. In comparison, the meteorological inputs were identified to have lower relevance for the proposed prediction targets. However, specific meteorological features were identified as having a significant influence on the prediction targets for the model region of this study, including outside air temperature, with roughly 6–10%, and ground temperature, with about 4–10% of the overall predictor relevance. The remaining meteorological features should not be entirely excluded from consideration either, as their relevance may vary across different regions.

This work suggests that the new prediction targets require individual feature engineering and selection processes. Most research combining district heating networks and ML approaches focuses on modeling the heat load. For this prediction target, the outside air temperature is determined as the most relevant predictor. In comparison, the outside air temperature shows less significance for the herein-suggested prediction targets.

Comparing the results in dependence on the application of recurrent or non-recurrent feature evaluation and selection methods, the approach resting on an RNN proves a surplus of information to be gained, e.g., from the higher dynamics of the outside temperature. With the first set of non-recurrent methods, only for the heating period and the target of the supply temperature, the highest impact of the outside temperature could be confirmed. Yet, in this specific case, depending on the applied method, the ground temperature can hold almost as much (around 8% for the supply temperature as the prediction target) or even more predictive power (up to 10%) for the targets of volume flow and return temperature.

The study also reveals that individual building behaviors vary, suggesting that averaging over several buildings can enhance the generalizability of conclusions across the district heating network. The investigation supports the notion that feature engineering plays a significant role in improving the processability of inputs. In this case, creating a feature for the heat flow and generating features from the time stamp proved especially beneficial. It further suggests separating the summer and the heating period in later modeling. An additional feature, structured as a uniform time series, can increase performance, depending on the model.

With a focus on increasing knowledge of energy systems, this study addresses the challenge of engineering and selecting relevant features for modeling user-level parameters in existing district heating networks. The findings suggest that modeling district heating networks requires a feature engineering and selection strategy customized for the specific district heating networks and prediction targets. The study presents a workflow and strategy for ML modeling at the user level in district heating networks. This aims to advance the sustainable and efficient operation of district heating networks as part of the global energy transition.

## Supplementary Information

Below is the link to the electronic supplementary material.

Supplementary Material 1

## Data Availability

Raw data for the model region dataset are not publicly available to preserve individuals’ privacy under the European General Data Protection Regulation. Anonymized datasets generated for this study are available from the corresponding author upon reasonable request and with the heat network operator’s consent.
