# Supplementary material for: Feature selection for specific prediction targets at the user level in a district heating network
Source: Sci Rep. 2025 Aug 14;15:29789. doi: 10.1038/s41598-025-15777-0 (PMC12354703; doi:10.1038/s41598-025-15777-0)
Supplement: Supplementary file 1 — Supplementary Material 1 [file 41598_2025_15777_MOESM1_ESM.docx]

Supplementary Information

Feature Selection for Specific Prediction Targets at the User Level in a District Heating Network

Samanta A. Weber¹^,^²*, Michael Fischlschweiger¹, Dirk Volta² & Jens Geisler²

¹ Chair of Technical Thermodynamics and Energy Efficient Materials Treatment, Institute for Energy Process Engineering and Fuel Technology, Clausthal University of Technology, 38678 Clausthal-Zellerfeld, Germany

² Energy and Life Science, University of Applied Sciences Flensburg, 24943 Flensburg, Germany

Corresponding Author

*Samanta A. Weber - https://orcid.org/0000-0002-6487-8248;
samanta.weber@tu-clausthal.de
samanta.weber@hs-flensburg.de

**Table S1** Cleaning methods applied to the data collected at the infeed facility

| Parameter at CHP | Treatment of outliers |
| --- | --- |
| Volume flow | Linear interpolation using mean; previous value using mean; previous value using moving mean with a 30 days window and a threshold factor of 2.5 |
| Supply temperature | Linear interpolation using mean, threshold factor 3.25; Linear interpolation using moving mean over 1-day window, threshold factor 2.5 |
| Return temperature | Linear interpolation using moving median over 71-day window, threshold factor 2.75 |
| Accumulated heat | Linear interpolation using moving mean over 14-day window, threshold factor 0.25; Linear interpolation using moving mean over 16 h-window, threshold factor 0.25 |
| Heat flow | Linear interpolation using moving median over 10 days-window, threshold factor 2.75 |
| Supply pressure | Linear interpolation using mean |
| Return pressure | Linear interpolation using mean |
| Moving mean | $MA=\frac{1}{k}\times\sum_{i=n-k+1}^{n} p_{i}$  n: last sample in considered window  k: window length (number of samples in window) |
| Moving median | $MM=median(p_{i}, p_{i+1}, \ldots, p_{n})$  With $i=n-k+1$  n: last sample in considered window  k: window length (number of samples in window) |

**Table S2** Cleaning methods applied to the meteorological data

| Meteorological parameter | Treatment of | |
| --- | --- | --- |
|  | Missing values | Outliers |
| Ground temperature | Linear interpolation with a maximum gap of 72 h | Moving median over 500 h and threshold factor of 3.5 |
| Precipitation | Linear interpolation with a maximum gap of 3 h | Linear interpolation using mean |
| Absolute moisture | Station 1: Linear interpolation with a maximum gap of 3 h Station 2: Nearest values | Station 1: Linear interpolation using moving median with an 18 h-window, threshold factor 8 Station 2: Linear interpolation using 24 days as window for the moving mean |
| Relative moisture | Station 1: Linear interpolation with a maximum gap of 3 h Station 2: Linear interpolation with a maximum gap of 3 h | Station 1: Linear interpolation using moving median with an 18 h- window, threshold factor 8 Station 2: Linear interpolation using mean, threshold factor 4.25 |
| Sun minutes | Linear interpolation with a maximum gap of 3 h | - |
| Wind speed | Linear interpolation with a maximum gap of 3 h | Station 1: Linear interpolation using mean, threshold factor 1 |
| Wind direction | Linear interpolation with a maximum gap of 3 h | Station 2: Nearest value using mean, threshold factor of 2.25 |


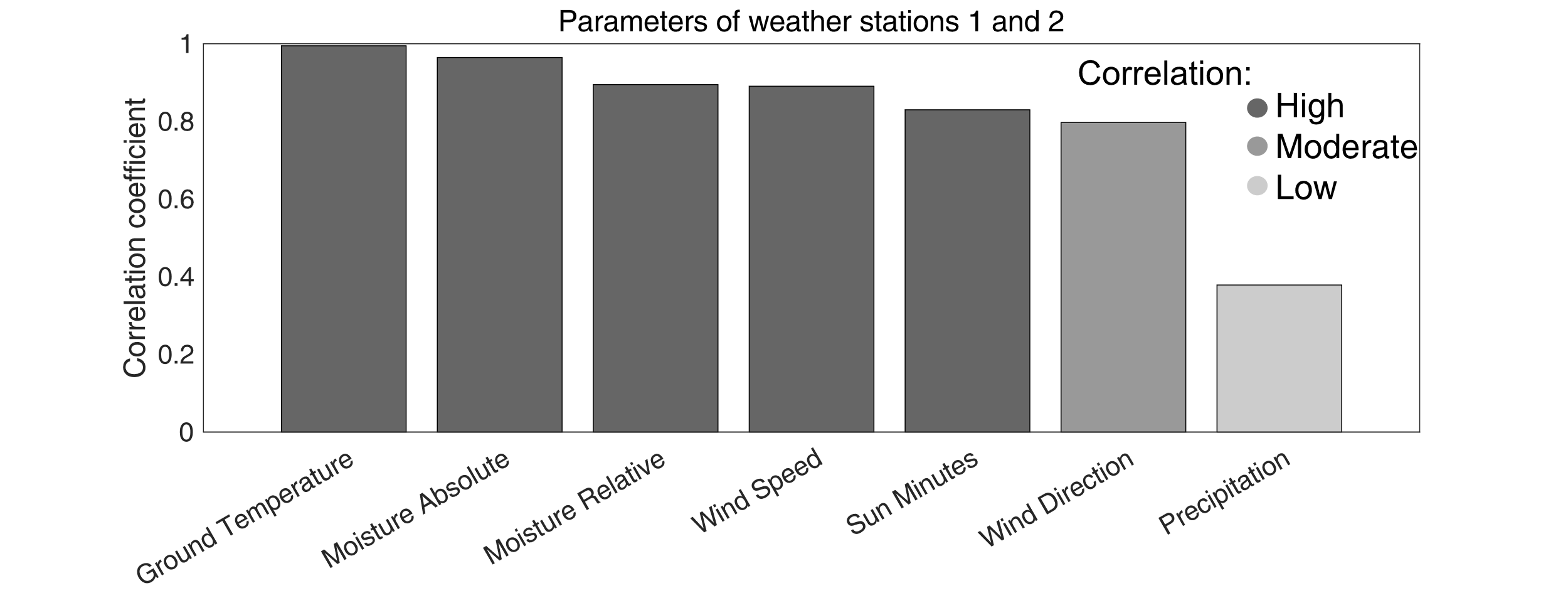


**Figure S1** Linear correlation coefficients of the parameters of weather stations 1 and 2. The generally high correlation of the data of both weather stations indicates that the applicability to the third location (model region) is given, apart from the data on precipitation showing a low correlation.


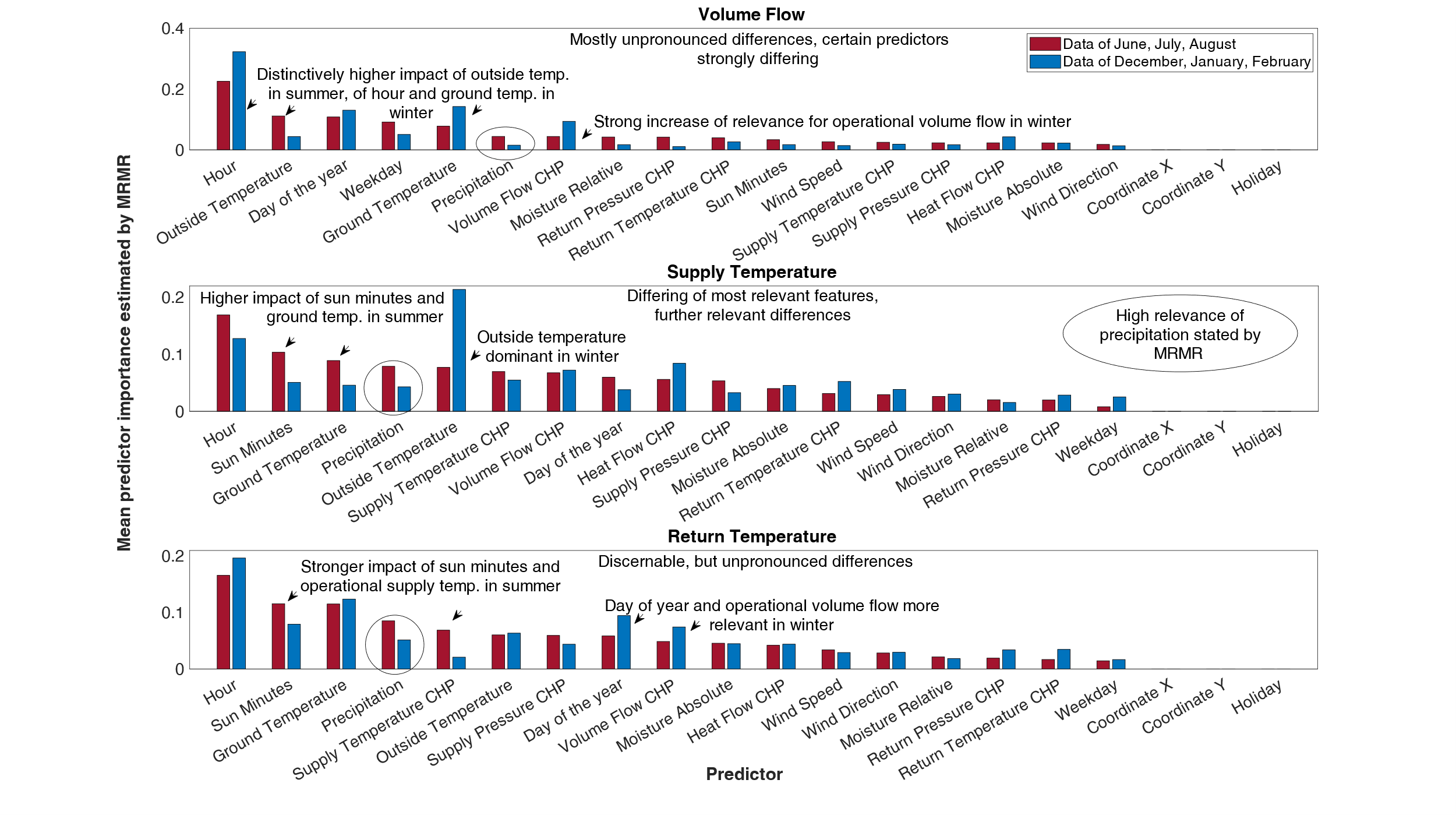


**Figure S2** Results for MRMR, summer, and winter separately. Comparing summer and winter data, the most relevant predictors differ in their evaluation, indicating the necessity to account for the seasons separately. The general order of relevance changes for specific features.
